# Hypothalamic control of brown adipose tissue thermogenesis

**DOI:** 10.3389/fnsys.2015.00150

**Published:** 2015-11-03

**Authors:** Sebastien M. Labbé, Alexandre Caron, Damien Lanfray, Boris Monge-Rofarello, Timothy J. Bartness, Denis Richard

**Affiliations:** ^1^Centre de Recherche de l’Institut Universitaire de Cardiologie et de Pneumologie de Québec, Department of Medicine, Université LavalQuébec, QC, Canada; ^2^Department of Biology, Center for Obesity Reversal (COR), Georgia State UniversityAtlanta, GA, USA

**Keywords:** brown adipose tissue, melanocortin, endocannabinoid, hypothalamus, steroidogenic factor 1, non-shivering thermogenesis

## Abstract

It has long been known, in large part from animal studies, that the control of brown adipose tissue (BAT) thermogenesis is insured by the central nervous system (CNS), which integrates several stimuli in order to control BAT activation through the sympathetic nervous system (SNS). SNS-mediated BAT activity is governed by diverse neurons found in brain structures involved in homeostatic regulations and whose activity is modulated by various factors including oscillations of energy fluxes. The characterization of these neurons has always represented a challenging issue. The available literature suggests that the neuronal circuits controlling BAT thermogenesis are largely part of an autonomic circuitry involving the hypothalamus, brainstem and the SNS efferent neurons. In the present review, we recapitulate the latest progresses in regards to the hypothalamic regulation of BAT metabolism. We briefly addressed the role of the thermoregulatory pathway and its interactions with the energy balance systems in the control of thermogenesis. We also reviewed the involvement of the brain melanocortin and endocannabinoid systems as well as the emerging role of steroidogenic factor 1 (SF1) neurons in BAT thermogenesis. Finally, we examined the link existing between these systems and the homeostatic factors that modulate their activities.

## Introduction

Brown adipose tissue (BAT) represents a major thermogenic effector. BAT is found in relative abundance in small mammals, where it plays a key role in thermoregulatory thermogenesis (Cannon and Nedergaard, 2004). Recent studies have confirmed not only its presence but also its functionality in adult humans (Cypess et al., 2009; Virtanen et al., 2009; van Marken Lichtenbelt et al., 2009; Ouellet et al., 2012), which has driven a renewed interest for the role of BAT in energy balance regulation in relation with obesity. There currently is a therapeutic interest in targeting BAT thermogenesis to treat excess fat deposition and related metabolic disorders (Chechi et al., 2014). One other aspect that supports a role of BAT in energy balance regulation is the involvement of brain neuronal circuits in the control of thermogenesis via the sympathetic nervous system (SNS; Bartness and Ryu, 2015; Chechi and Richard, 2015). Indeed, most circuits controlling BAT thermogenesis genuinely play roles in energy balance regulation (Richard and Picard, 2011; Chechi et al., 2013). BAT thermogenesis control is insured by different brain systems, essentially owing to the hypothalamus and the brainstem, that insure the autonomic control of BAT (Richard, 2015). This review briefly overviews the role of the thermoregulatory pathway and its modulation by energy balance systems for the purpose of thermogenesis. The review also addresses three important hypothalamic homeostasis regulatory pathways modulating BAT thermogenesis, which are the melanocortin and endocannabinoid systems, as well as the steroidogenic factor 1 (SF1; also known as NR5A1) neurons of the ventromedial hypothalamus (VMH). We further aim at clarifying how these systems interact and how homeostatic hormones such as insulin and leptin strategically modulate their activity.

### BAT Thermogenesis

BAT is a specialized tissue whose main function is to produce heat (Cannon and Nedergaard, 2004). It is present in small mammals, allowing them to produce non-shivering thermogenesis (NST) and therefore live in cold environments without relying on muscle-derived shivering thermogenesis (Cannon and Nedergaard, 2004). The thermogenic potential of BAT owes to the presence of uncoupling protein 1 (UCP1), a protein uniquely found in the inner membrane of the brown adipocyte numerous mitochondria, that uncouples substrate oxidation from electron transport. In rodents, BAT is “classically” located in the interscapular, subscapular, axillary, perirenal, and periaortic regions (Cannon and Nedergaard, 2004), while in humans, it is found in subscapular, cervical, peri-spinal, mediastinal, periaortic, pericardial and periadrenal regions (Cannon and Nedergaard, 2004; Lidell et al., 2014). The thermogenic potential of BAT is remarkable, making it the primary site of NST in rodents (Foster, 1974; Depocas et al., 1978; Foster and Frydman, 1978). Impressively this tissue can account for up to 75% of the increased metabolic rate induced by noradrenaline (NA) in cold-adapted animals (Foster, 1984). Importantly, BAT can burn up to 50% of ingested triglycerides and 75% of ingested glucose (Nedergaard et al., 2011). We also recently estimated that the BAT intracellular triglycerides pools contributed to up to 84% of thermogenesis during an acute cold challenge (Labbé et al., 2015). These estimations demonstrate the undeniable ability of BAT to regulate systemic substrates homeostasis.

Histologically, BAT cells differ from white adipose tissue (WAT) adipocytes, the latter being considered as mainly fat reservoirs containing few mitochondria and a single large lipid droplet. Brown adipocytes, which can also develop in WAT in a process referred to as “beiging” of WAT (Wu et al., 2012a, 2013) contain numerous small lipid vacuoles surrounded by numerous well-developed mitochondria (Lim et al., 2012), which contain iron pigmented-cytochromes that are largely responsible for the brownish color of BAT. Previous investigations (Timmons et al., 2007; Seale et al., 2008; Tseng et al., 2008) have provided compelling evidence that brown and white fat cells are not only distinct histologically, but that they also origin from different precursor cells (Shan et al., 2013; Sanchez-Gurmaches and Guertin, 2014a,b). Accordingly, BAT cells in so-called classical brown fat depots (listed above) share their origin with myogenic-factor 5 (Myf5)-expressing cells, which are also precursors of myocytes (Crisan et al., 2008; Kajimura and Saito, 2013). For comprehensive overviews on the transcriptional mechanisms that control adipose tissues development, we referred the reader to other excellent reviews (Harms and Seale, 2013; Liu et al., 2013; Wu et al., 2013; Sanchez-Gurmaches and Guertin, 2014b; Sidossis and Kajimura, 2015).

### SNS Control of Brown Adipose Tissue

BAT thermogenesis is highly dependent on the SNS (Bartness et al., 2010; Bartness and Ryu, 2015). In fact, brown adipocytes are richly innervated as evidenced by thyroxin hydroxylase positive cells (Murano et al., 2009), and also highly expressed the β3-adrenoreceptor, a Gs protein-coupled receptor primarily involved in BAT thermogenesis (Cannon and Nedergaard, 2004; Bartness et al., 2010; Richard and Picard, 2011). The release of NA by SNS efferent postganglionic fibers and the binding of this transmitter to the β3-adrenergic receptors (mostly) lead to a cascade of metabolic events triggering unimpeded substrate oxidation and ultimately heat production (Bachman et al., 2002; Jimenez et al., 2002; Lowell and Bachman, 2003). Mechanistically, the release of NA enhances brown adipocyte thermogenic activity (heat production as such) by increasing cyclic adenosine monophosphate (cAMP) levels, which in turn activates the protein kinase A (PKA) and the ultimate generation (through lipolysis) of fatty acids that serve as energy substrate and UCP1 activators (Cannon and Nedergaard, 2004). In addition, the enhanced SNS activity also increases BAT thermogenic capacity (number of brown adipocytes, quantity of mitochondria per adipocyte, expression of UCP1 and accompanying thermogenic proteins; Cannon and Nedergaard, 2004; Nedergaard and Cannon, 2013). Importantly, conditions such as cold exposure (cold-induced thermogenesis) and feeding (diet-induced thermogenesis) are integrated by the central nervous system (CNS) in order to stimulate brown adipocyte thermogenesis. In addition, β3-adrenergic agonism, be it driven by cold or drugs, also promotes the “beiging” of white fat, which currently represents a major focus of attention in among investigators addressing the brain control of thermogenesis (Oldfield et al., 2002; Contreras et al., 2014; Bartness and Ryu, 2015; López et al., 2015).

**Figure 1:**
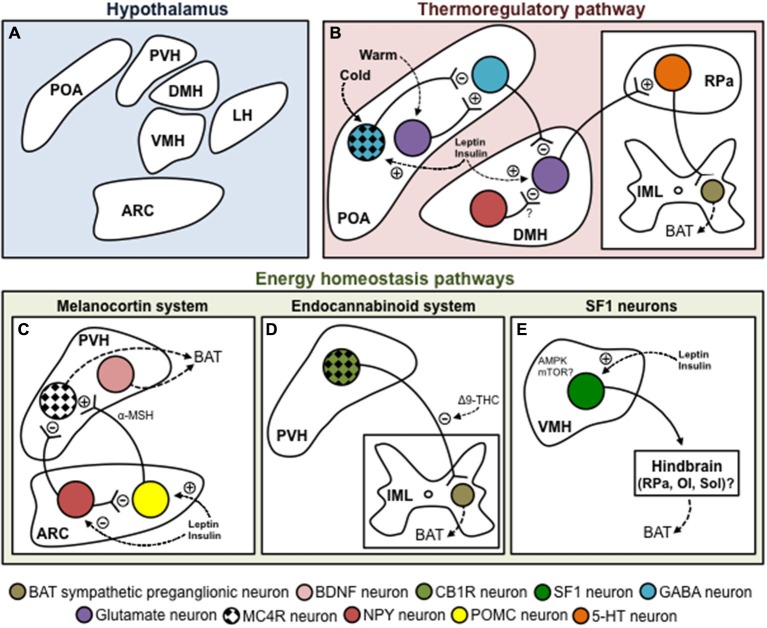
**Hypothalamic control of brown adipose tissue thermogenesis. (A)** Schematic and tentative representation of the hypothalamic structures involved in the control of BAT thermogenesis. **(B)** POA is considered to be a major coordinator of thermoregulation as it receives inputs from thermoreceptors essentially located in the skin. Within the POA, cold appears to signal mainly through the MnPO, where GABAergic neurons inhibit GABAergic neurons of the MPO. On the other hand, warm activates glutamatergic neurons in the MnPO, which activate GABAergic neurons of the MPO. One important relay receiving inputs from the POA to regulate BAT metabolism is the DMH. The GABAergic neurons of the MPO provide a negative tonic inhibition toward glutametergic neurons located in the DMH. DMH neurons project to the rostral ventromedial medulla, apparently to the RPa, amain site of BAT SNS premotor neurons. NPY neurons of the DMH could apparently also negatively affect BAT thermogenesis, possibly by inhibiting glutamatergic neurons located in the DMH itself. There also exists at least three important energy homeostasis pathways for BAT thermogenesis. **(C)** The melanocortin system is a critical component for the maintenance of energy balance. In the ARC, NPY/AgRP neurons inhibit, while POMC neurons (through the production of α-MSH) activate MC4R neurons located in the PVH. These neurons stimulate BAT thermogenesis. However, their identity is actually unknown. Non-MC4R neurons expressing BDNF were also recently shown to regulate BAT metabolism. **(D)** The endocannabinoid system represents another important system regulating BAT thermogenesis. We have recently shown that injection of Δ9-THC into the fourth ventricle blunts the thermogenic effects of MTII injected in the PVH. This suggests that MC4R-CB1R neurons located in the PVH regulate BAT metabolism. **(E)** SF1 neurons of the VMH are also well known to affect BAT thermogenesis. Converging evidence indicates that both AMPK and mTOR signaling are molecularly involved in this fine regulation. The VMH neurons regulating thermogenesis seem to relay through hindbrain structures such as the RPa, OI or Sol. **(C–E)** It is noteworthy that in addition to interacting together, these hypothalamic systems regulating BAT thermogenesis are modulated by important homeostatic hormones such as leptin and insulin. Abbreviations: 5-HT, serotonine; α-MSH, α-melanocyte-stimulating hormone; Δ9-THC, Δ9-tetrahydrocannabinol; AMPK, AMP-activated protein kinase; ARC, arcuate nucleus; BAT, brown adipose tissue; BDNF, brain-derived neurotrophic factor; CB1R, cannabinoid receptor type 1; DMH, dorsomedial hypothalamus, GABA, gamma-aminobutyric acid; IML, intermediolateral nucleus; LH, lateral hypothalamus; MC4R, melanocortin receptor 4; MnPO, median preoptic area; MPO, medial preoptic area; MTII, melanotan II; mTOR, mechanistic target of rapamycin; NPY, neuropeptide Y; OI, inferior olive; POA, preoptic area; POMC, proopiomelanocortin; PVH, paraventricular hypothalamus; RPa, raphe pallidus; SF1, steroidogenic factor 1; Sol, nucleus of the solitary tract; VMH, ventromedial hypothalamus.

The determination of the brain regions genuinely driving the SNS outflow to BAT has been a challenging issue. In that regard, retrograde transneuronal viral tracing using the pseudorabies virus (PRV) have been of paramount importance in delineating the brain regions as well as the neuronal circuits connected to BAT and WAT (Bartness et al., 2005; Song et al., 2005). When injected into BAT, PRV is retrogradely transported to the brain via the SNS outflow, allowing for the identification of the origin of the neuronal pathways connected to this tissue (Bamshad et al., 1999; Song et al., 2008). These experiments demonstrated that the SNS activity to BAT is governed by brain regions involved in the regulation of body temperature (Nakamura and Morrison, 2008) as well as in the regulation of energy balance (Richard, 2015). In addition to the cold-activated pathways (e.g., the thermoregulatory pathway), a number of hypothalamic nuclei have been linked to the control of BAT thermogenesis to allow diet-induced thermogenesis (e.g., the energy homeostasis regulatory pathway). These nuclei especially include the arcuate nucleus (ARC), preoptic area (POA), dorsomedial hypothalamus (DMH), paraventricular hypothalamus (PVH), lateral hypothalamus (LH) and ventromedial hypothalamus (VMH; Richard, 2015; Figure 1A).

## The Thermoregulatory Pathway

The brain integration of information about external temperature is insured by a complex neurocircuitry that includes several brain nuclei. Within the hypothalamus, the POA is considered to be a major coordinator of thermoregulation as it receives inputs from thermoreceptors essentially located in the skin (Osaka, 2004; Nakamura and Morrison, 2008, 2011; Morrison et al., 2014; Figure 1B). The POA comprises the median preoptic area (MnPO), the medial preoptic area (MPO), the lateral preoptic area (LPO) and the preoptic periventricular area (POP). Skin or direct POA cooling (Magoun et al., 1938; Hammel et al., 1960; Imai-Matsumura et al., 1984; Martelli et al., 2014) induces SNS activation of BAT in laboratory rodents. Within the POA, cold appears to signal mainly through GABA neurons to ultimately release an inhibition on the DMH neurons triggering thermogenesis (Morrison et al., 2014). Furthermore, the POA projects to other hypothalamic nuclei known to control thermogenesis, including the VMH (Hogan et al., 1982; Imai-Matsumura et al., 1984; Preston et al., 1989) and PVH (Horn et al., 1994; Caldeira et al., 1998; Zhang et al., 2000). For a comprehensive overview on the central neural pathways for thermoregulation, we referred the reader to excellent reviews by Morrison et al. (2008, 2012, 2014).

One important relay receiving inputs from the POA to regulate adipose tissues metabolism is the DMH. The DMH plays a wide range of metabolic and behavioral function, including body weight regulation (Bellinger and Bernardis, 2002). Stimulation and disinhibition of DMH neurons, by parenchymal microinjection of respectively glutamate and GABA_A_ receptor antagonist, increases BAT thermogenesis and elevates body core temperature (Figure 1B; Zaretskaia et al., 2002; Cao et al., 2004; Dimicco and Zaretsky, 2007; Morrison and Nakamura, 2011). A recent study also identified a subset of DMH cholinergic neurons (Jeong et al., 2015), whose activity was elevated by warm ambient temperature and associated with a decrease in BAT activity and body temperature (Jeong et al., 2015). Importantly, DMH lesion abolishes the ability of external cooling or direct stimulation of the POA to stimulate BAT thermogenesis (Hogan et al., 1982; Preston et al., 1989; Monge-Roffarello et al., 2014a), reinforcing the fact that POA activation of BAT involves the DMH. Also, consistent with the fact that the DMH is an intermediate relay for POA-dependent thermogenesis, neuronal tracing studies have identified the POA as the region containing the largest number of neurons retrogradely labeled from the DMH (Thompson and Swanson, 1998). Many neurons in the POA are known to be GABAergic (Okamura et al., 1990), and those that are relevant to thermoregulation are thought to exert tonic inhibition of downstream DMH neurons important for BAT activation (Chen et al., 1998). Microinjection of bicuculline methiodide, a GABA_A_ receptor antagonist, is an efficient means of evoking activation of neurons in the DMH that are involved in the SNS control of BAT, suggesting that these neurons are restrained by tonic GABAergic inhibition under normal circumstances. Thus, GABAergic neurons in the POA are likely to provide a key source of tonic inhibitory input to sympathoexcitatory neurons in the DMH. It is important to note that DMH neurons do not directly project to BAT SNS preganglionic neurons. Instead, DMH neurons project to the rostral ventromedial medulla, apparently to the RPa, the main site of BAT SNS premotor neurons (Nakamura et al., 2005; Yoshida et al., 2009). Consequently, the RPa could represent an essential component of this POA-DMH thermoregulatory pathway.

Several groups have reported that *neuropeptide Y* (*NPY*) gene expression increases in the DMH of obese animals (Kesterson et al., 1997; Guan et al., 1998; Tritos et al., 1998; Bi et al., 2001) or in animals with increased energy demands (Bi et al., 2003; Kawaguchi et al., 2005). DMH-NPY overexpression leads to hyperphagia and obesity (Yang et al., 2009), while DMH-NPY knockdown protects against high-fat diet-induced obesity (Chao et al., 2011). DMH-NPY knockdown also causes increased expression of UCP1 in BAT (Chao et al., 2011). In support to this observation, DMH-NPY knockdown results in an increase in BAT thermogenesis as directly determined by a rise in BAT temperature (Chao et al., 2011). At room temperature, BAT temperature is significantly higher in rats having a DMH-NPY knockdown (Chao et al., 2011). These data support a role for DMH-NPY in modulating BAT thermogenesis and in affecting energy expenditure. Moreover, knockdown of DMH-NPY in rats was reported to increase the thermogenic response following 6 h of cold exposure (6°C; Chao et al., 2011). Supporting these results, it was demonstrated that NPY production as well as the number of NPY-immunoreactive fibers decreased in the DMH during an acute cold exposure context (2 h at 4°C), possibly to facilitate BAT thermogenesis (Park et al., 2007). Considering all these evidences for a negative role of DMH-NPY neurons on BAT thermogenesis, it is possible that the function of these neurons is to inhibit the glutamatergic neurons located in the DMH itself (Figure 1B).

## Hypothalamic Energy Homeostasis Pathways of BAT Thermogenesis

In addition to the thermoregulatory thermogenesis, the activity of BAT might also be modulated by neurons that are part of hypothalamic energy homeostasis regulatory pathways located mainly in the ARC, PVH, LH and VMH (Chechi et al., 2013; Stefanidis et al., 2014; Richard, 2015). These neurons are sensitive to energy balance fluctuations and could also act independently of the POA-DMH-RPa thermoregulatory circuit as in the case of PVH neurons directly projecting to the IML (Zheng et al., 1995). Over the last decades, it has become clear that the melanocortin and endocannabinoid systems, as well as the SF1 neurons of the VMH play a pivotal role in the hypothalamic energy homeostasis regulatory pathways of thermogenesis.

### The Melanocortin System in the Control of BAT Thermogenesis

The brain melanocortin system is a critical component for the maintenance of energy balance (De Jonghe et al., 2011; Xu et al., 2011; Richard, 2015). It primarily consists of neurons producing melanocortins and agouti-related peptide (AgRP) together with neurons upon which the melanocortins and AgRP act and which express the melanocortin receptors 3 (MC3R) and 4 (MC4R). AgRP has been referred to as a melanocortin-receptor inverse agonist (Ollmann et al., 1997) and more recently as a possible “biased” agonist (Ghamari-Langroudi et al., 2015). The activation of the melanocortin system essentially emerges from the ARC, which represents one of the most acknowledged hypothalamic nuclei in energy homeostasis (Schwartz et al., 2000). The ARC includes at least two known populations of neurons strongly involved in the regulation of energy balance. One population co-synthesizes, together with AgRP, NPY and gamma-aminobutyric acid (GABA), while the other synthesizes pro-opiomelanocortin (POMC; Morton et al., 2006; Dietrich and Horvath, 2013; Mercer et al., 2013). These neurons abundantly project to the neuroendocrine and metabolic divisions of several brain nuclei including the PVH, which constitutes an important relay for the ARC neurons involved in energy balance regulation. POMC neurons release α-melanocyte-stimulating hormone (α-MSH), a peptidergic fragment produced following POMC cleavage. α-MSH has emerged, in rodents at least, to be the melanocortin the most involved in energy balance. Within the brain, α-MSH binds to MC4R, which likely represents the main melanocortin receptor in energy balance regulation (Butler, 2006; Ellacott and Cone, 2006; De Jonghe et al., 2011; Xu et al., 2011). The melanocortin system has been linked to both energy expenditure and food intake and thus likely represents a genuine regulator of energy balance (Richard, 2015).

Early studies showing that numerous brain populations of MC4R expressing-neurons are (poly)synaptically connected to BAT, have contributed to underline the relevance of the melanocortin system in the metabolic control of BAT activity (Song et al., 2008). Consistently, pharmacological investigations have established that the central injection of the melanocortin receptors agonist melanotan II (MTII) directly into the PVH leads to BAT thermogenesis (Figure 1C; Song et al., 2008; Monge-Roffarello et al., 2014b). The functional significance of this system in regulating BAT metabolic activity has also been validated in *Pomc* knock-out (KO) and *Mc4r-*KO mice, which exhibit widespread obesity resulting from both a hyperphagic and hypometabolic phenotype (Butler and Cone, 2002). Consistently, this phenotype is characterized by a decrease in cold-induced thermogenesis (Butler et al., 2001; Voss-Andreae et al., 2007).

*Mc4r* mRNA is found in oxytocin positive cells (Siljee et al., 2013), suggesting that oxytocin neurons could be good candidates to connect MC4R signaling to BAT activation. In that respect, retrograde transneuronal viral tracing experiments performed in rodents have also demonstrated that BAT is (poly)synapticaly connected to caudal PVH neurons expressing oxytocin (Oldfield et al., 2002). It has also been shown that the deletion of oxytocin or its receptor impaired BAT activation induced by cold exposure (Kasahara et al., 2007) or high-fat diet (Wu et al., 2012b), suggesting a direct involvement of this population of PVH neurons in BAT regulation. However, a recent report indicate that rescuing MC4R expression in neither oxytocin nor corticotropin-releasing hormone (CRH)-expressing neurons rescue food intake or energy expenditure in *Mc4r-KO* mice (Shah et al., 2014). This observation tends to exclude the direct involvement of these two neuronal populations in the PVH MC4R signaling. Moreover, this study also excludes the direct involvement of PVH vasopressin- and prodynorphin-expressing neurons in MC4R-dependent BAT activation (Shah et al., 2014). Interestingly, another study recently reported that brain-derived neurotrophic factor (BDNF) neurons in the PVH control both food intake and energy expenditure (An et al., 2015). Accordingly, *BDNF* expression in the PVH was reported to be increase following cold exposure, whereas its specific ablation impairs BAT thermogenesis (Figure 1C; An et al., 2015). However, BDNF neurons of the PVH did not expressed MC4R, thus suggesting that the thermogenic effects of MC4R in the PVH occur in another unidentified population.

The POA represents an important hypothalamic area expressing the MC4R. It is noteworthy that in hamsters no less than 77% of the 589 neurons (some 450 neurons), which are (poly)synaptically connected to interscapular BAT, express MC4R (Song, 2005; Song et al., 2008). This group of cells represents the second largest population of MC4R neurons in the hypothalamus that are connected to interscapular BAT (Song, 2005; Song et al., 2008). Recently, we have demonstrated that pharmacological activation of MC4R (using MTII) in MPO increases BAT temperature, CO_2_ production and BAT non-esterified fatty acids (NEFA) uptake measured by ^14^C-bromopalmitate (Figure 1B; Monge-Roffarello et al., 2014a). This further suggests an important relation between the thermoregulatory circuit and the melanocortin system in the regulation of BAT thermogenesis. These data also suggest a potential involvement of MC4R in cold-induced thermogenesis. Accordingly, it was shown that the acute effects of cold exposure seem to be mediated by MC4R, since mice lacking MC4R exhibit a defect in BAT UCP1 expression after 4 h at 4°C (Voss-Andreae et al., 2007). However, it is important to mention that UCP1 in normally induced following a longer cold exposure in *Mc4r-*KO mice, suggesting that melanocortin system-mediated NTS is differentially modulated by chronic cold or that alternative compensatory mechanisms develop. A recent study also demonstrated the importance of MC4R in cholinergic autonomic pre-ganglionic neurons in terms of regulation of cold-induced thermogenesis and glucose homeostasis, reinforcing the importance of MC4R in systemic metabolism, even in extra-hypothalamic area (Berglund et al., 2014).

Supporting a relation between the melanocortin and thermoregulatory pathways, our group have also recently demonstrated that kainic acid lesion of the DMH blunts the thermogenic effects of MPO activation by MTII (Figure 1B; Monge-Roffarello et al., 2014a). Accordingly, we showed that the DMH lesion blunts the increase of BAT temperature and NEFA uptake, as well as the induction of thermogenic genes such as *Dio2* and *Pgc1α*, induced by the MTII injection in the MPO of male rats (Monge-Roffarello et al., 2014a). Our data supported previous findings suggesting that DMH could be involved in the modulation of the melanocortin system activation through BAT (Enriori et al., 2011). It is conceivable that MC4R-MPO activation of BAT is mediated by a DMH relay in line with the thermoregulatory network described in the previous section (Morrison et al., 2014).

### The Endocannabinoid System in the Control of BAT Thermogenesis

Another important player in the hypothalamic energy homeostasis regulatory pathway of thermogenesis appears to be the endocannabinoid system. The CNS endocannabinoid system includes neurons expressing the cannabinoid receptor type 1 (CB1R). CB1R is one of the two identified cannabinoid receptors and is expressed in energy-balance brain structures (Richard et al., 2009) that produce or inactivate N-arachidonoyl ethanolamine (anandamide) and 2-arachidonoylglycerol (2AG), the most abundantly formed and released endocannabinoids (Di Marzo and Matias, 2005). It is well recognized that the endocannabinoid system activation reduces energy expenditure as administration of the cannabinoid receptor agonist Δ^9^-tetrahydrocannabinol (Δ9-THC) inhibits BAT thermogenesis (Verty et al., 2011). Reliably, subchronic administration of rimonabant, a CB1R antagonist, has been shown to enhance thermogenesis (Verty et al., 2009), while this stimulatory effect was totally lost after denervation of BAT (Bajzer et al., 2011). However, the mechanisms whereby endocannabinoids affect energy homeostasis are still unclear. Growing evidence nonetheless suggests an interaction with the melanocortin system. Accordingly, we recently demonstrated that the stimulatory effect of MTII injection into the PVH on BAT temperature was blocked by the co-injection of Δ9-THC in the fourth ventricle (Figure 1D; Monge-Roffarello et al., 2014b). These results indicate that the endogenous endocannabinoids could, via possibly a pre-synaptic effects exerted at the level of the IML (or brainstem), inhibit the stimulating effects of the PVH MC4R-containing neurons driving BAT activation. Consistently it has been shown that hindbrain overexpression of the hydrolase monoacylglycerol lipase (MGL), which can inactivate 2AG, leads to an increase in BAT activity (Jung et al., 2012), further supporting a role of the hindbrain CB1R neurons in BAT thermogenesis.

### The Steroidogenic-Factor 1 Neurons in the Control of BAT Thermogenesis

The VMH has been implicated in a wide array of physiological and behavioral processes since the classical lesion studies of Hetherington and Ranson (Hetherington and Ranson, 1940; Canteras et al., 1994; King, 2006). Several studies have in fact confirmed that VMH lesions affect both body weight and food intake (reviewed in Choi et al., 2013). In the 80’s, many studies have supported the role of the VMH in the SNS control of BAT. Convincingly, it was shown that the electrical stimulation of the VMH increased BAT temperature in rats (Perkins et al., 1981; Holt et al., 1987; Hugie et al., 1992). VMH lesions/electrical stimulation were further demonstrated to affect BAT NE turnover, indicative of a mobilization of the autonomic nervous system (Yoshida and Bray, 1984; Saito et al., 1987). Although these studies pointed out the relationship between VMH and BAT thermogenesis, it was only recently that the molecular events responsible for this thermogenic function were investigated (Choi et al., 2013). This was made possible by the discovery of SF1, an important target for VMH-specific genetic manipulations.

The fact that CNS SF1 is restrictedly expressed in VMH neurons has been exploited in the development of many VMH-specific transgenic models (Bingham et al., 2006; Dhillon et al., 2006). *Sf1-*KO mice revealed SF1 to be essential in establishing the cytoarchitecture of the VMH (Ikeda et al., 1995; Shinoda et al., 1995). As SF1 deletion led to neonatal lethality due to adrenal insufficiency (SF1 is also expressed in adrenal glands and gonads; Majdic et al., 2002), different approaches were used to develop viable SF1 models. By transplanting adrenal glands into *Sf1*-KO animals, neonatal lethality was circumvented, and obesity was observed (Majdic et al., 2002). Accordingly, investigators developed a post-natal VMH-specific *Sf1-*KO model, using CamKII-Cre line, and observed a similar phenotype explained by an impaired energy expenditure along with a decrease in BAT UCP1 expression (Kim et al., 2011). Therefore, the discovery of SF1 has shed light on the many facets of the SNS control of BAT by the VMH. In recent years, the accessibility of the SF1-Cre model has extended our understanding of the VMH-dependent hormonal regulation of BAT.

How the VMH stimulation couples the SNS outflow to BAT remains obscure. In that regard, it is noteworthy that retrograde transneuronal viral tracing studies have not revealed the VMH as a main hypothalamic structure in driving the sympathetic outflow to BAT activation (Bamshad et al., 1999). It has nonetheless been suggested that the RPa and inferior olive could act as relays for the VMH neurons to modulate the SNS outflow to BAT (Morrison, 1999). SF1 neurons in the VMH are also known to project to several autonomic centers, including the nucleus of the solitary tract (Sol; Lindberg et al., 2013; Figure 1E). This raises a particular interest in the involvement of brainstem structures in the regulation of BAT thermogenesis, as Sol neurons have been shown to integrate several metabolic signals influencing BAT activity (reviewed in Morrison et al., 2014). Combining retrograde transneuronal viral tracing and the use of SF1-cre lines could be a potential strategy to identify missing links between the VMH and BAT thermogenesis.

A role for the endocannabinoid system in SF1-dependent regulation of BAT activity was also suggested following the observation that CB1R mRNA was highly expressed in the VMH (Richard et al., 2009), and that the deletion of CB1R in the hypothalamus led to an increase in energy expenditure (Cardinal et al., 2012). Supporting this possibility, a recent study in which CB1R was ablated in SF1-expressing neurons demonstrated the importance of the VMH endocannabinoid system in determining the metabolic adaptation to various dietary conditions (Cardinal et al., 2014). Although no difference was observed in terms of energy expenditure, this study did not invalidate the potential involvement of VMH CB1R neurons in regulating BAT thermogenesis since prenatal recombination was used to generate the model. Growing evidence suggests that the traditional genetic modifications that occur prenatally may lead to compensatory mechanisms (Choi et al., 2013). In that regards, it was recently showed that the expression pattern of SF1 differs between prenatal and adult mice (Cheung et al., 2013). The generation of an adult inducible SF1 model could be instrumental in understanding the role of SF1 neurons on the different metabolic variables that they affect.

## Homeostatic Modulation of the Regulatory Systems Involved in BAT Thermogenesis

The adipocyte-derived hormone leptin is a key regulator of energy balance as it acts in the brain to decrease food intake and increase energy expenditure (Gautron and Elmquist, 2011). As such, leptin is considered as a prominent catabolic hormone, since disruption of the gene coding for leptin (*ob/ob* mice) and/or its receptor (*db/db* mice) induces massive obesity, resulting from hyperphagia and reduced BAT thermogenesis (Thenen and Mayer, 1976; Leiter et al., 1983; Malik and Young, 1996; Mizuno et al., 1998; Goncalves et al., 2009). In the CNS, insulin also acts as a catabolic hormone through stimulating energy expenditure and reducing food intake (Menéndez and Atrens, 1991; Varela and Horvath, 2012). The following section described how these important homeostatic hormones affects BAT thermogenesis, by acting on the different regulatory systems and pathways that we have been described.

### Homeostatic Modulation of the Melanocortin System in the Control of BAT Thermogenesis

Investigations aimed at identifying the ARC neurons involved in BAT metabolism regulation have established that leptin receptors (LEPR)-expressing neurons are (poly)synaptically connected to BAT (Oldfield et al., 2002). In addition, the activities of POMC and NPY/AgRP/GABA neurons have long been considered to be directly modulated by energy reserve sensing hormones, such as leptin (Varela and Horvath, 2012). Moreover, leptin is ineffective in stimulating thermogenesis in *Pomc*-KO and *Mc4r-*KO mice, reinforcing the notion that the melanocortin system is a major relay for leptin-induced thermogenesis (Butler and Cone, 2002). Although the role of leptin in mediating BAT thermogenesis is well acknowledged, the molecular signaling pathway allowing LEPR to activate BAT was only recently investigated. One important candidate could be the phosphatidyl inositol 3-kinase (PI3K), which is a downstream effector of both the leptin and insulin receptors signaling. However, deletion of PI3K in POMC neurons does not appear to lead to widespread obesity and does not affect long term whole body weight regulation (Hill et al., 2008). A recent study also revealed that POMC-specific deletion of rho-associated protein kinase 1 (ROCK-1), leads to mild adiposity, suggesting that leptin signaling in these neurons involves ROCK-1 activation (Huang et al., 2012). Besides, ROCK-1 ablation in AgRP neurons was also shown to increase O_2_ consumption, suggesting that this protein is also involved in leptin-induced inhibition of AgRP neurons (Huang et al., 2013). However, ROCK-1 disruption in these neuronal populations only induces mild obesity as compared to the massive obesity observed in mice lacking LEPR in ARC GABAergic neurons (Vong et al., 2011). Another study revealed the importance of an intact leptin signaling for the regulation of adipose tissues metabolism (Dodd et al., 2015). Indeed, ablation of both protein tyrosine phosphatases PTP1B and TCPTP, two important negative regulators of leptin signaling, in POMC neurons, increased *UCP1* expression and temperature in BAT (Dodd et al., 2015).

### Homeostatic Modulation of the VMH SF1 Neurons in the Control of BAT Thermogenesis

VMH integrates many hormonal signals that could modulate SNS-mediated BAT thermogenesis. There is evidence suggesting that leptin signaling in the VMH plays a pivotal role in the mediation of the SNS tone; injection of leptin into the VMH increases glucose uptake in various tissues including BAT, an effect that is blunted by the sympathetic denervation (Haque et al., 1999; Minokoshi et al., 1999; Toda et al., 2009). The recent generation of SF1-Cre transgenic lines allowed to further understand the mechanisms whereby leptin acts in the VMH to control BAT thermogenesis (Xu et al., 2010; Kim et al., 2011, 2012). Even if signal transducer and activator of transcription 3 (STAT3) signaling plays a major role in leptin’s control of energy metabolism (Bates et al., 2003), recent attention has focused on the implication of the PI3K signaling pathway in mediating hypothalamic leptin actions (Hill et al., 2008). Accordingly, LEPR and PI3K expression in SF1 neurons were both proven to be required for normal body weight homeostasis (Dhillon et al., 2006; Bingham et al., 2008; Xu et al., 2010). Leptin was also shown to directly activate SF1 neurons since selective deletion of the LEPR from these neurons induced obesity (Balthasar et al., 2004; Dhillon et al., 2006; Bingham et al., 2008). In addition, mice with reduced PI3K activity in the VMH proved to be more sensitive to diet-induced obesity due to a reduced energy expenditure (Xu et al., 2010). Altogether, these observations indicate an important role for PI3K signaling in SF1 neurons in mediating the effects of leptin on BAT thermogenesis.

PI3K is also phosphorylated and activated following insulin receptor (IR) activation, and consequently plays a major role in insulin function, mainly through the activation of protein kinase B (Akt/PKB; Laplante and Sabatini, 2012). Forkhead box O1 (FoxO1) is a well-characterized transcriptional factor that target Akt/PKB. Once activated, Akt/PKB phosphorylates FoxO1, preventing its translocation to the nucleus. Hence, Akt/PKB reduces the transcriptional activity of FoxO1. Interestingly, mice lacking FoxO1 in SF1 neurons are lean due to an increased energy expenditure (Kim et al., 2012). These findings indicate a likely crosstalk between leptin and insulin signaling pathways in the regulation of BAT thermogenesis by the VMH. However, further studies are needed to understand the entire molecular cascade and the different contributors of this leptin and insulin relationship. For example, the mechanistic target of rapamycin (mTOR), which is downstream of PI3K-Akt signaling, could play an important role in SF1-mediating effects on energy homeostasis. mTOR nucleates two distinct multi-protein complexes termed mTORC1 and mTORC2 that play important roles in metabolism (Laplante and Sabatini, 2012; Caron et al., 2015). Interestingly, it was shown that leptin deficiency suppresses VMH mTORC1 activity (Villanueva et al., 2009) and that PI3K-mTORC1 signaling is critical in mediating the SNS effects of leptin (Harlan et al., 2013). In addition, mTORC2 neuronal deficiency reduces energy expenditure and core temperature by impairing hypothalamic Akt/PKB signaling (Kocalis et al., 2014). It is noteworthy that we recently demonstrated a high expression of the mTOR endogenous regulator DEP-domain containing mTOR-interacting protein (Deptor) in the dorsomedial part of the VMH, which houses the SF1-expressing neurons (Caron et al., 2014). The demonstration of the potential involvement of Deptor in the control of BAT thermogenesis by the VMH awaits further investigation.

Another important molecular target of the homeostatic regulation of BAT thermogenesis is AMP-activated protein kinase (AMPK). As such, it was recently revealed that AMPK activity in the VMH mediated the effects of both thyroid hormone and estradiol on BAT SNS activity and the resulting thermogenesis (López et al., 2010; Martínez de Morentin et al., 2014). Reinforcing the importance of VMH AMPK in regulating BAT thermogenesis, ablation of bone morphogenetic protein 8B (BMP8B) in mice impairs thermogenesis and reduced metabolic rate, at least by reducing hypothalamic AMPK activity (Whittle et al., 2012). It was also recently showed that central injection of a glucagon-like peptide 1 (GLP-1) receptor agonist in mice stimulates BAT thermogenesis by activating hypothalamic AMPK (Beiroa et al., 2014). Finally, studies also indicate that nicotine increases energy expenditure by modulating the hypothalamic AMPK-BAT axis (Martínez de Morentin et al., 2012; Seoane-Collazo et al., 2014).

### Homeostatic Modulation of the Thermoregulatory Pathway

Leptin also contributes to BAT sympathetic outputs through GABAergic and glutamatergic neurons of the POA (Zhang et al., 2011; Rezai-Zadeh and Münzberg, 2013). It is well known that leptin-deficient mice are hypothermic and are unable to defend their body temperature during an acute cold exposure (Trayhurn et al., 1976, 1977). The thermoregulatory defects in these mice have been attributed to defective BAT thermogenesis (Goodbody and Trayhurn, 1981, 1982), but little is known about the neuronal circuits that are involved. Interestingly, exogenous leptin injection rapidly rescues their ability to survive during a cold exposition (Trayhurn et al., 1976; Ukropec et al., 2006), indicating that leptin *per se* is necessary for a normal thermoregulatory response. Recently, it was shown that LEPR neurons in the POA, as well as in the DMH, represent a potential component of the thermoregulatory circuit (Zhang et al., 2011). These data strongly suggest that LEPR neurons in both the POA and the DMH represent thermoregulatory effectors that play a key role in the regulation of BAT thermogenesis (Nakamura et al., 2005, 2009). Confirming these results, neuronal activation of DMH LEPR neurons promotes BAT thermogenesis in mice (Rezai-Zadeh et al., 2014).

Less is known about the impact of insulin and its receptor in thermoregulatory thermogenesis. However, a study has shown that injection of insulin in the POA increases BAT thermogenesis through IR-expressing warm sensitive neurons (Sanchez-Alavez et al., 2010). Insulin seems to hyperpolarize warm-sensitive neurons and to decrease firing rates of these neurons through the DMH. Whether PI3K signaling is involved is unknown, but considering the evidence reported, it is possible that PI3K connects the effects of both leptin and insulin in POA-dependent thermogenesis. This, however, needs to be further substantiated.

## Concluding Remarks

Understanding how the brain controls BAT thermogenesis is an important prerequisite to fully comprehend the metabolic role that this tissue plays in energy homeostasis. We have overviewed in this short article some of the main systems and circuits that control BAT thermogenesis with a particular focus on the melanocortin and endocannabinoid systems as well as on the SF1 neurons. We have also tried to clarify how all these systems and circuits interact together and how they are influenced by different peripheral homeostatic signals. Finally, we have briefly addressed some potential pathways coupling the metabolic and thermoregulatory neuronal circuits driving BAT thermogenesis.

## Funding

DR is supported by the Canadian Institutes of Health Research (CIHR) and the Natural Sciences and Engineering Research Council of Canada (NSERC). AC holds a fellowship from the CIHR Training Program in Obesity/Healthy Body Weight. SML holds a CIHR postdoctoral fellowship.

## Conflict of Interest Statement

AC, SML, DL, BMR, TJM and DR have nothing to disclose. The authors declare that the research was conducted in the absence of any commercial or financial relationships that could be construed as a potential conflict of interest.
